# Prevention of social exclusion and role of antenatal care by BRAC community health workers in improving safe motherhood and neonatal care in urban slums of Bangladesh

**DOI:** 10.1371/journal.pone.0235340

**Published:** 2020-07-08

**Authors:** Saira Parveen Jolly, Tridib Roy Chowdhury, Mahfuzar Rahman, Ariful Alam, Kaosar Afsana

**Affiliations:** 1 Research and Evaluation Division, BRAC, Dhaka, Bangladesh; 2 Health, Nutrition and Population Programme, BRAC, Dhaka, Bangladesh; Erasmus Medical Center, NETHERLANDS

## Abstract

The transformation of the BRAC MANOSHI programme from humanitarian to a social enterprise model, has made it increasingly urgent to enumerate the minimum number of door-to-door antenatal care (ANC) visits by community health workers (CHWs), for the purpose of effectively improving facility delivery. Thus prevent social exclusion of poor slum communities in Bangladesh with regard to safe motherhood and essential newborn care (ENC). This cross-sectional study was conducted, during March–July, 2015 in slums of Chittagong, Dhaka and Sylhet city corporations of Bangladesh. A census was conducted among 25,700 households covering 10 branch offices of MANOSHI to identify women with a delivery outcome in the preceding three years of the survey. A total of 1100 respondents were interviewed randomly through a structured questionnaire. These women were stratified into three categories-1, 2 & 3, consisting of 497, 205 and 398 women respectively. Women in category-1 did not receive any ANC checkup from the BRAC CHWs, while women in category-2 and category-3 received one to three and ≥four ANC checkups from BRAC CHWs respectively. Data was analysed using STATA Version 13 (Chicago Inc.). Findings revealed that women, who received ≥four ANC checkups from BRAC CHWs, are 25% more likely to avail facility delivery [adjusted Prevalence Ratio (aPR) 1.25; 95% confidence interval (CI) (1.01–1.54)] compared to the women who did not receive any ANC from BRAC CHWs. Women in category-2 [aPR3.64; 95% CI (1.76–7.54)] and in category-3 [aPR5.92; 95% CI (3.04–11.53)] respectively had four and six folds higher tendency to receive postnatal care (PNC) within 48 hours after delivery. Furthermore, facility delivery improved PNC assisted by medically trained providers (MTPs) within 48 hours after delivery and ENC in both categories 2 & 3. The evidence shows that at least four ANC visits of BRAC CHWs can increase institutional delivery, and which can further facilitate PNC and ENC visits. At present, the BRAC MANOSHI programme needs to implement feasible strategies to include pregnant women in the slums in receiving at least four ANC checkups by BRAC CHWs for ensuring safe motherhood and newborn care.

## Introduction

The World Health Organization (WHO) recommends that all pregnant women should have at least eight high quality antenatal care (ANC) checkups during their pregnancy [1]. However, earlier recommendation of the same organization was to provide at least four ANC checkups during pregnancy, which has been followed by BRAC MANOSHI programme in Bangladesh since 2007 [2]. An ANC checkup has its own significance in improving birth preparedness, enabling women to identify and treat illnesses during pregnancy as well as in increasing use of emergency obstetric care (EmOC) facilities. As a consequence, ANC checkup reduces the risk of both maternal and neonatal mortality [2–6]. Evidence shows that at least four ANC checkups has a positive impact in increasing the rate of facility delivery, which ultimately facilitates rapid reduction of maternal and neonatal mortality [6–9].

In many developing countries, Millennium Development Goal (MDG)-5 for reduction of maternal mortality had not been achieved due to disparities between rich and poor, lack of proper utilization of continuum of care starting with ANC, skilled assisted delivery through postnatal care (PNC) during the entire pregnancy and postpartum period [4,10–12]. It is obvious that poor women lack in a continuum of care during pregnancy and childbirth. Globally, every year 3,030,000 women die because of various maternal morbidities [4]. The marginalized, poor women across the world having limited access to health facilities and die from postpartum, heamorrhage, which could have been prevented through intervention of complication readiness, skilled assisted delivery, and PNC [4,13]. On the other hand, women from affluent group die due to complications such as abortion, ectopic pregnancy and miscarriage [4]. The post-2015 agenda on sustainable development goal (SDG) targeted reduction of inequity in addressing maternal and child mortality [10]. Thus, an immediate implementation of maternal and child healthcare services has become an urgent need for the poor.

A facility based continuum of obstetric care would be the best practice for sustained reduction in maternal and neonatal mortality [14]. However, in order to make services accessible to the resource-poor setting, an integrated approach that includes elevated health system with supply of services, community-based intervention, home visitations by community health workers (CHWs) and community mobilization for improved services, is required [15,16]. The Government of Bangladesh has already taken such initiatives to improve the health system that would ultimately lead to achieving MDG-4 and 5. This Government, jointly with the United Nations and non-government organisations (NGOs), has upgraded district and sub-district hospitals across Bangladesh for comprehensive and basic emergency obstetric care (EmOC) [17,18], introduced the health voucher scheme [19,20] and implemented maternal and neonatal health programme called the Maternal and Neonatal Health Initiatives in Bangladesh (MNHIB) [21]. Besides, a large number of CHWs are working through the doorstep approach to improve maternal health [22]. In cities, the Government has partnered with NGOs to implement the maternal, neonatal and child health (MNCH) care services at community level [23]. Furthermore, private facilities are rapidly proliferating in cities [24]. Despite, all these efforts, people living in slums are lagging behind in terms of using MNCH care services compared to non-slum areas in the cities [25]. Most of the residents of slums are migrating from rural areas. They neither can afford health services nor they are well-informed of different urban health facilities [26,27]. Furthermore, they prefer unskilled assisted home delivery compared to hospital delivery, which has led to a higher maternal and neonatal mortality [25,28,29]. In addition, women in slums, engaged in market employment, has less chance of receiving adequate maternal healthcare services [30]. Moreover, maternal deaths in Bangladesh have been stalled for a decade and slums could be the pocket in urban areas where women have limited access to maternal healthcare services [31]. Therefore, a provision of affordable and accessible healthcare services is necessary to eliminate existing health barriers of the slum population.

To address the issue, since 2007, BRAC, the largest NGO in Bangladesh, has been working to implement a community-based MNCH care service package called MANOSHI, targeting slum population across cities in Bangladesh [32,33]. This programme can potentially improve utilization of maternal and neonatal healthcare services and reduce neonatal deaths [33,34]. After confirming pregnancy, BRAC CHWs provide women monthly ANC checkups including physical examination; on-spot biochemical examination for blood glucose, blood grouping and urinary albumin; counseling for birth preparedness and complication readingness (BPCR), food and nutrition, facility delivery and essential newborn care (ENC) free of cost. The CHWs refer pregnant women to the nearby EmOC facilities when complications occur. During PNC visits, BRAC CHWs examine health conditions of both the mother and her neonate, ensuring thermal and cord care to prevent infection. Each slum within the study areas is equipped with either a BRAC maternity center (BMC) or a BRAC delivery center (BDC) for preventing unsafe home delivery at an affordable charge. In BMC and BDC, deliveries are conducted by midwives and urban birth attendants respectively and are allowed to conduct only normal vaginal delivery along with a provision for episiotomy. In these facilities, midwives also provide paid ANC and PNC checkups. Both facilities are supervised by MBBS doctors. This programme has strong referral linkages with EmOC facilities. Mothers with complications are referred to these referral facilities to save lives of both mothers and their babies.

Recently, BRAC Health, Nutrition and Population programme (HNPP) including MANOSHI programme is undergoing a transformation from a philanthropic model to a social enterprise (SE) model to enable the programme to be a self-sustaining entity instead of being a donor dependent agency. In addition, since Bangladesh is progessing economically and at the same time people are influxing in the slums due to rapid urbanization, this situation is causing an immense pressure on the urban health system [25,26]. However, BRAC will not change their mission and vision of helping the marginalized population. BRAC HNPP will countinue its MNCHcare services in exchange of minimal charges from the poor community. This strategy is called the social enterprise model. This social entrepreneurship can help non-profit organizations operate in an innovative way [35]. Business experts believe that when traditional resources reduce constantly and competition for these common resources becomes extremely high, it becomes urgent for NGOs to employ professional business operations and marketing techniques to improve the quality of products and efficiency in services so as to serve the community better [35]. They also argue that in order to achieve this, a change is required in attitude, approach, behaviour and ultimately in the culture of the non-profit sector, as only the fittest (enterprising non-profits) will survive the increased competition over scarce public and private money, as resource scarcity and resource mobilization theories suggest [35]. BRAC has generated some key organizational factors including addressing specific social and client needs, and has introduced a unique ‘BRAC Model’ and strategies for their SEs, visionary leadership and competent management with proper organizational foundation to assist the organization in its capacity to become a sustainable and successful SE. According to BRAC, a social enterprise is a business venture that aims to achieve financial returns while fulfilling social, environmental, and/or other developmental goals [36].

Through these services the beneficiaries would receive similar ANC and PNC services from the BRAC CHWs as before. The BRAC CHWs would counsel about BPCR and safe institutional delivery either at BRAC delivery/maternity centre or at other public/private institutions for saving lives of both mothers and neonates. Incorporating user fees might reduce ANC service utilization by poor women, as we assume that receiving multiple paid ANC checkups from BRAC CHWs would make it unaffordable to them [37]. In addition, the repeated migration of slum communities would increase women’s risk to be excluded from the services of BRAC CHWs during their pregnancy [25,28]. BRAC MANOSHI programme is trying to ahieve SDG-3 to reduce all preventable maternal and neonatal deaths. Usually, the BRAC CHWs visit a pregnant woman once in a month. In the current situation, the programme would not compromise its social impact and ensure the facility delivery for all women for sustained reduction of maternal and neonatal mortality [32]. Thus, a policy including a minimum number of ANC visits and a councelling package for BRAC CHWs to ensure institutional delivery, PNC and ENC has became urgent. Therefore, this study aimed to identify the minimum number of ANC visits of BRAC CHWs that would be effective in elevating utilization of in-facility delivery, PNC and ENC among marginalized people of urban slums in Bangladesh.

## Methodology

**Fig 1:**
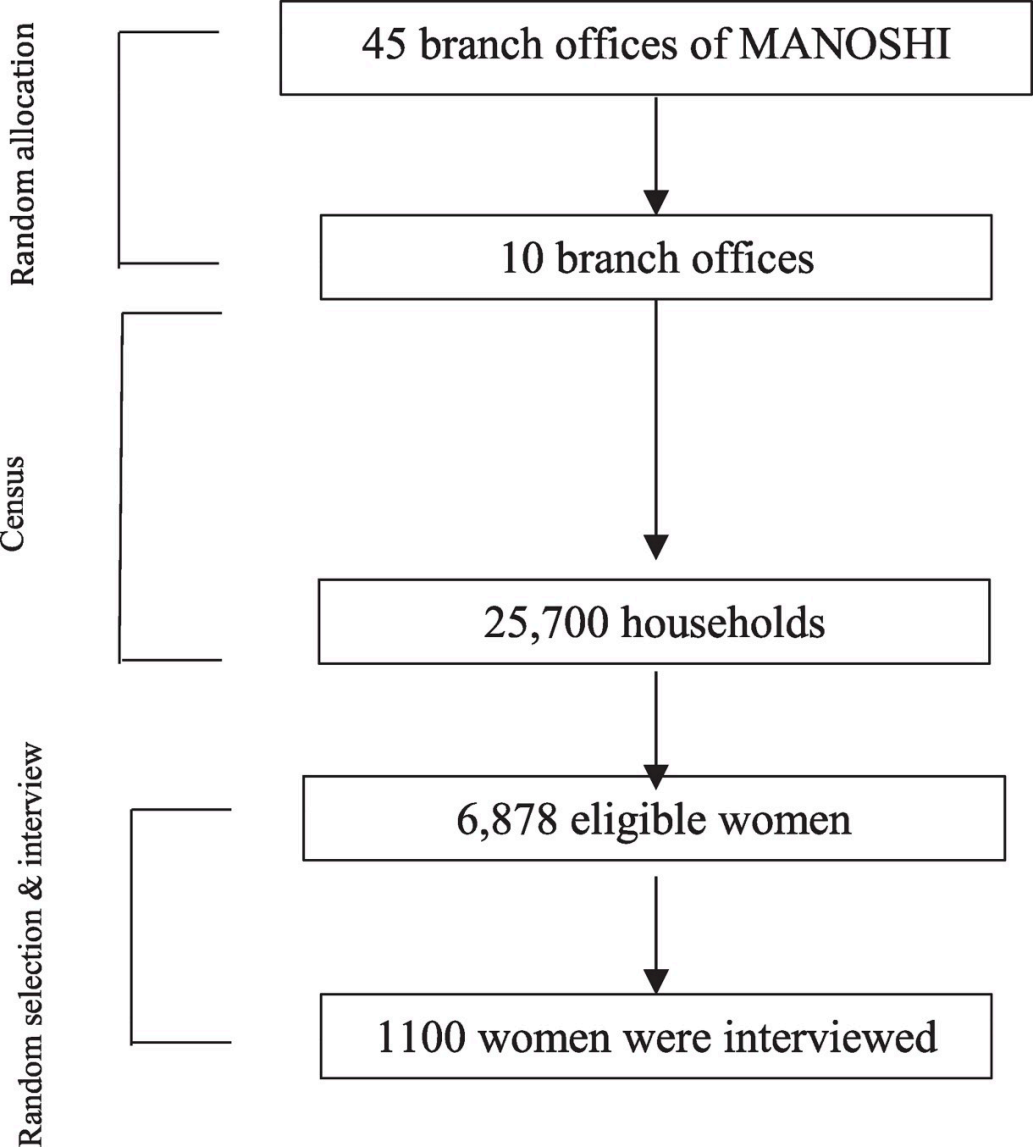
Sampling and randomization for selecting the respondents.

A community based cross-sectional study was conducted, from March to July 2015, in ten MANOSHI branch offices including *Mogbazar*, *Pallavi-10*, *Cantonment*, *Sarulia*, *Sutrapur*, *Sabuzbagh* of Dhaka City; *Jalalabad*, *Kotowali*, *Cononnel Hut* of Chittagong City; and *Sylhet upashohor* of Sylhet City. Slums surrounded all branch offices and six of them were equipped with BMCs, and four had BDCs. Married women aged 15–49 years having a delivery outcome in the preceding three years of the survey were included in the study. To get a precision of facility delivery in the previous three years in urban areas of Bangladesh (49.5%) [25], with 95% confidence level, 5% precision and 5% contingency the required sample size for this study was 806 women. Out of 45 branch offices in Dhaka, Chittagong and Sylhet, 10 were selected randomly (Fig 1). Initially, a census was conducted in 2500 households from each slum. A total of 25,700 households were included in the census. We found 6,878 households with an eligible woman. Finally, 1100 women were randomly interviewed from 10 slums.

### Census and survey

Two structured questionnaires were developed. One was for census and the other was for survey. We used a questionnaire that had been used in an earlier national level survey and in the earlier study of MANOSHI programme [33,34]. In the survey, we captured the maternal and neonatal information and only included the most relevant questions consistent with the objective of the study. After that, we conducted a pilot study to check the feasibility of the questionnaire including the sequence of events, familiarity of the interviewers with the questions and interview scenario, duration, cost and any adverse event while asking sensitive questions. In addition, to check the consistency, responses of reinterviews were entered and analysed to examine the correlation between the two responses.

Skilled female interviewers having previous experience on maternal health survey and male supervisors were recruited. Ten teams, consisting of a supervisor and four interviewers, were formed. Initially, they received a two-day training including one-day field-test followed by a feedback session for procedures of administering the census. The census was conducted for a month and data was entered and coded. Later ten sets of random numbers were generated separately for each slum to select 110 women. Before the survey, a seven-day training was organized. A training manual was developed to guide the interviewers during interviews. Another field-test was conducted during training session in the neighboring slums of the study areas to check inter-observer variation followed by a feedback session. In person, interviews were carried out for data collection. Each data form was crosschecked twice by the supervisor and another team member. Three field operation officers and an investigation team were based at study sites for quality control. They checked interviews randomly, data forms onspot and re-interviewed 10% of mothers within two days after an interview. Regular meetings with field staffs and quality control teams were held at the MANOSHI branch office to address problems and share the new experiences.

### Ethical approval

BRAC Research and Evaluation Division (RED) approved the proposal following existing rules. Before the interview, field enumerators explained to each respondent about the nature of the programme, rationale of the study, questionnaire, confidentiality and the risks and benefits associated with the study in the presence of a witness. Once they voluntarily agreed, they were asked to provide their signature or thumb impression.

### Data analysis

Analysis of parametric continuous variables was performed using one-way ANOVA and results were depicted as Mean±SD and P-value. All the categorical variables were analyzed using chi-square (χ^2^) test and results were expressed as percentage, number and P-value. A wealth index based on the ownership of household assets is widely recognised as a proxy for household economic status [33]. In order to get a wealth index, data regarding some categorical variables such as, property, household assets, household construction materials, water, sanitation, and fuel supply were collected dichotomously [33]. Later, factor analysis was used to assign weighing values to indicator variables. The wealth quintile was constructed using the rank procedure. The association between indicators and predictors was analyzed by cox regression analysis, and through robust variance estimation. The data was expressed in adjusted Prevalence Ratio (aPR) with 95% confidence interval (CI). The aim of this analysis was to explore the number of ANC checkups and ensure the validity of other indicators. In a cross-sectional study, output of logistic regression ‘odds ratio’ overstates the relative risk. On the other hand, cox regression with robust variance gives the best estimate of relative risk in a cross–sectional study. Therefore, cox regression analysis is considered as the most appropriate analytical tool for estimating relative risk [38,39]. The exposure variable, ANC from BRAC CHWs, was stratified into three categories based on the number of ANC check-ups. Category-1, 2 and 3 comprise: no ANC, one to three ANCs and ≥four ANC check-ups respectively. Therefore, these three ANC categories were the independent variables whereas, institutional delivery, seeking treatment for delivery complications, PNC within 48 hours and ENC were the dependent variables. An ***ANC visit*** refered to check-up done by a healthcare provider during pregnancy. In addition, *PNC* was defined as the care of the mother after childbirth until about six weeks and ***ENC*** was a set of newborn care practices for preventing hypothermia and sepsis of neonates. The most important ENC practices recommended to be followed include cutting cord by sterile blade, tying cord with sterile thread, wiping immediately after birth with dry cloth, wraping from head to toe with dry cloth and initiation of breastfeeding within one hour after birth. An ***institutional delivery*** was defined as a delivery conducted in a facility by a doctor, nurse, midwife, paramedic, and family welfare visitor (FWV). A ***medically trained provider (MTP)*** included a qualified doctor, nurse, midwife, paramedic, FWV and a community skilled birth attendant (CSBA), while a ***trained providers*** included MTP, BRAC SK and urban birth attendant (UBA).

All outcome and predictor variables were also stratified. Dummy variables were generated and avalue of “0” was given for reference otherwise; the value “1” was used. Analysis was performed using STATA Version 13 (Chicago Inc.). Significance was taken at p<0.05.

## Results

After stratification, the numbers of ANC visits by BRAC CHWs, in category-1, 2 and 3 were 497, 205 and 398 respectively. Table 1 depicts that the average age and age at first marriage and conception among women in category-1 were significantly higher compared to women in category-2 and 3. In category-3 women had bigger family size, completed at least secondary level of schooling and were wealthier compared to categories 1 and 2. More than 60% of women in the three categories reported availing obstetric care facilities within 0.5 km of their locality. A similar proportion of women in three categories had pregnancy, intrapartum and postpartum complications.

**Table 1:** Comparison of socio-demographic and reproductive history of the respondents by number of ANC received from BRAC CHWs.

Variables	Category	Number of ANC checkups from BRAC CHWs						P-value
		None		One -three		≥four		
		Category 1		Category 2		Category 3		
N		497		205		398		
Average age, in year, Mean ± SD**		26.35	±5.52	25.01	±4.48	25.76	±5.58	0.010
Average age at first marriage, in year, Mean ± SD**		17.45	±3.01	17.00	±2.70	16.86	±2.67	0.028
Average age at first conceive, in year, Mean ± SD**		19.16	±3.38	18.45	±2.92	18.43	±3.15	0.041
Religion, % (n) *	Islam	94.16	(468)	92.20	(189)	87.69	(349)	0.002
	Others ^b^	5.84	(29)	7.80	(16)	12.31	(49)	
Primary occupation, % (n) *	Housewife	91.75	(454)	94.63	(194)	92.96	(370)	0.398
	Others ^a^	8.25	(41)	5.37	(11)	7.04	(28)	
Involved in earning, % (n) *		19.92	(99)	17.56	(36)	19.60	(78)	0.764
Family type, %(n) *	Conjugal	37.42	(186)	40.00	(82)	33.67	(134)	0.267
	Nuclear	62.58	(311)	60.00	(123)	66.33	(264)	
Number of parity, % (n) *	0–1	42.45	(211)	44.39	(91)	40.20	(160)	0.446
	2	35.21	(175)	32.68	(67)	32.41	(129)	
	≥3	22.33	(111)	22.93	(47)	27.39	(109)	
Average household size, Mean±SD**		5.07	±2.32	5.06	±2.18	5.11	±2.03	0.018
Year of schooling, % (n) *	None	14.08	(70)	22.44	(46)	24.37	(97)	0.000
	Primary incomplete	16.70	(83)	22.93	(47)	22.86	(91)	
	Primary complete	12.27	(61)	15.61	(32)	15.33	(61)	
	Secondary incomplete	32.80	(163)	32.20	(66)	30.90	(123)	
	Secondary +	24.14	(120)	6.83	(14)	6.53	(26)	
Wealth index, % (n) *	Poorest	14.89	(74)	25.37	(52)	23.62	(94)	0.000
	Second	17.51	(87)	20.98	(43)	22.61	(90)	
	Middle	16.10	(80)	21.46	(44)	24.12	(96)	
	Fourth	19.52	(97)	20.00	(41)	20.60	(82)	
	Richest	31.99	(159)	12.20	(25)	9.05	(36)	
Distance of facility with obstetric care, % (n) *	<0.5 km	63.58	(316)	65.37	(134)	68.34	(272)	0.328
	≥0.5 km	36.42	(181)	34.63	(71)	31.66	(126)	
Result of last delivery, % (n) *	Live birth	98.79	(491)	100.00	(205)	99.75	(397)	0.090
	Still birth	1.21	(6)	0.00	(0)	0.25	(1)	
Had complication, % (n) *	During last pregnancy^d^	57.75	(287)	56.09	(115)	54.02	(215)	0.536
	During last delivery^e^	37.42	(186)	33.17	(68)	40.95	(163)	0.168
	During postpartum period of last delivery^f^	25.4	(126)	26.8	(55)	25.4	(101)	0.910

^a^ Handicraft, day labourer (non-agri), service, small business, beggar, maid servant, tailor, teacher

^b^Hindu, Christian, Buddhist

^c^Pregnancy complication defined as having any of one symptoms of high blood pressure, oedema, convulsion, excessive bleeding, high fever, severe headache, blurry vision, reduced/absent fetal movement, lower abdominal pain, anaemia, jaundice, excessive vomiting, diabetes

^d^Delivery complication defined as having any of the symptoms of hand/leg prolapsed, convulsion, mother fainted, high fever, perineal tear, nuchal cord, retained/ruptured placenta, severe headache, excessive bleeding, blurry vision, high blood pressure, obstructed labour, prolonged labour, mal-position

^e^Postpartum complication defined as having any of the symptoms of high blood pressure, blurry vision, severe headache, convulsion, high fever, foul smelling discharge, excessive bleeding, oedema, jaundice, lower abdominal pain

*p value was calculated using Chi-Square test

**p value was calculated using One-way ANOVA

Table 2 shows that the proportion of utilization of modern contraceptive methods, one or ≥four ANC checkups from MTPs, institutional delivery, delivery at private clinics and C-section were highest in category-1 compared to other two categories (p<0.001). In contrast, home delivery was higher among women in category-2 (40.44% vs 55.12% vs 45.72%), while more women in category-3 delivered their babies at BMCs (4.02% vs. 13.66% vs. 15.83; p<0.001) and BDCs (0.60% vs 7.31% vs 12.06%; p<0.001). About 55.94%, 32.68% and 39.70% women in category 1, 2 and 3 respectively received PNC from MTP within 48 hours after delivery (p<0.001). PNC checkups from BRAC CHWs within 48 hours after delivery, among the women who had home delivery, were higher in category-3 compared to category 1 and 2 (30.46% vs 5.77% and 19.01%; p<0.001). Seeking treatment against complications during pregnancy, delivery and postnatal period among the three categories were comparable. A significantly higher proportion of women in category-3 reported of having received all ENC services from trained providers for their newborns compared to the other two categories (62.80% vs 53.92% vs 69.77%; p<0.01).

**Table 2:** Maternal and essential newborn care services received by the women and their neonates.

Variables	Types	Number of ANC checkups from BRAC CHWs						P-value*
		None		One -three		≥four		
		Category 1		Category 2		Category 3		
N		497		205		398		
		%	n	%	n	%	n	
**Maternal health care services**								
Contraceptive prevalence rate		74.45	(370)	84.39	(173)	84.67	(337)	0.000
Use of modern contraceptive methods ^a^		68.20	(339)	75.60	(155)	77.89	(310)	0.003
Received at least one ANC from MTP ^b^		84.31	(419)	67.32	(138)	62.31	(248)	0.000
Received four or more ANCs from MTP ^b^		50.50	(251)	20.98	(43)	10.80	(43)	0.000
Institutional delivery		59.56	(296)	44.88	(92)	55.28	(220)	0.002
	Public hospital	13.28	(66)	9.75	(20)	19.09	(76)	0.000
	Private clinic	35.81	(178)	16.58	(34)	13.61	(54)	
	BMC	4.02	(20)	13.66	(28)	15.83	(65)	
	Other NGO linic	6.44	(32)	4.89	(10)	6.28	(25)	
Home delivery		40.44	(201)	55.12	(113)	45.72	(178)	
	Home	39.84	(198)	47.81	(98)	32.66	(130)	0.000
	BDC	0.60	(3)	7.31	(15)	12.06	(48)	
Skilled /MTP ^b^ assisted delivery		62.37	(310)	46.34	(95)	58.08	(231)	0.000
Mode of delivery	Normal	53.12	(264)	71.32	(146)	69.60	(277)	0.000
	Episiotomy	9.66	(48)	8.78	(18)	10.30	(41)	
	C-section	37.22	(185)	20.00	(41)	20.10	(80)	
Received PNC from MTP ^b^ within 48 hrs after delivery		55.94	(278)	32.68	(67)	39.70	(158)	0.000
Received PNC from BRAC CHW within 48 hrs after delivery		2.41	(12)	11.21	(23)	15.07	(60)	0.000
Received PNC from BRAC CHW within 48 hrs after home delivery		5.77	(12)	19.01	(23)	30.46	(60)	0.000
Received treatment against	ANC complications	83.62	(240)	77.39	(89)	81.39	(175)	0.640
	Delivery complications	90.32	(168)	92.64	(63)	92.63	(151)	0.847
	Post-delivery complications	83.33	(105)	74.54	(41)	74.25	(75)	0.274
**Essential newborn care services**								
N^c^			490		204		397	
Received all ENCforneonates		48.77	(239)	39.22	(80)	50.88	(202)	0.022
Individual ENC performed	Wiping	97.97	(482)	100.0	(204)	98.49	(391)	0.126
	Wrapping with warm clothes	71.95	(354)	66.18	(135)	68.51	(272)	0.267
	Initiation of breast milk within one hour	72.56	(357)	68.63	(140)	75.57	(300)	0.188
	Cutting cord by sterile blade ^d^	94.31	(464)	93.14	(190)	97.98	(389)	0.007
	Tying cord by sterile thread ^e^	88.01	(433)	78.43	(160)	88.92	(353)	0.001
ENC received fromtrained providers (qualified doctor/ nurse/FWV/BRAC CHW/UBA)		62.80	(309)	53.92	(110)	69.77	(277)	0.001

^a^Modern contraceptive methods includes pill, condom, intrauterine device, injectable, implant, ligation, vasectomy

^b^MTP: Medically Trained Provider includes qualified doctor, nurse, FWV, midwife, paramedics

^c^Six respondents were not interviewed on neonatal health as they had stillbirth

^d^ Sterile blade includes surgical blade, delivery kit’s blade, new blade and boiled, new blade and hit up

^e^ Sterile tread includes thread boiled & delivery kit’s thread

* *p* value was calculated using Chi-Square test

ANC-Antenatal Care; PNC-Postnatal Care; ENC-Essential newborn care; UBA- Urban birth attendant

BMC- BRAC maternity centre

BDC- BRAC delivery centre

**Fig 2:**
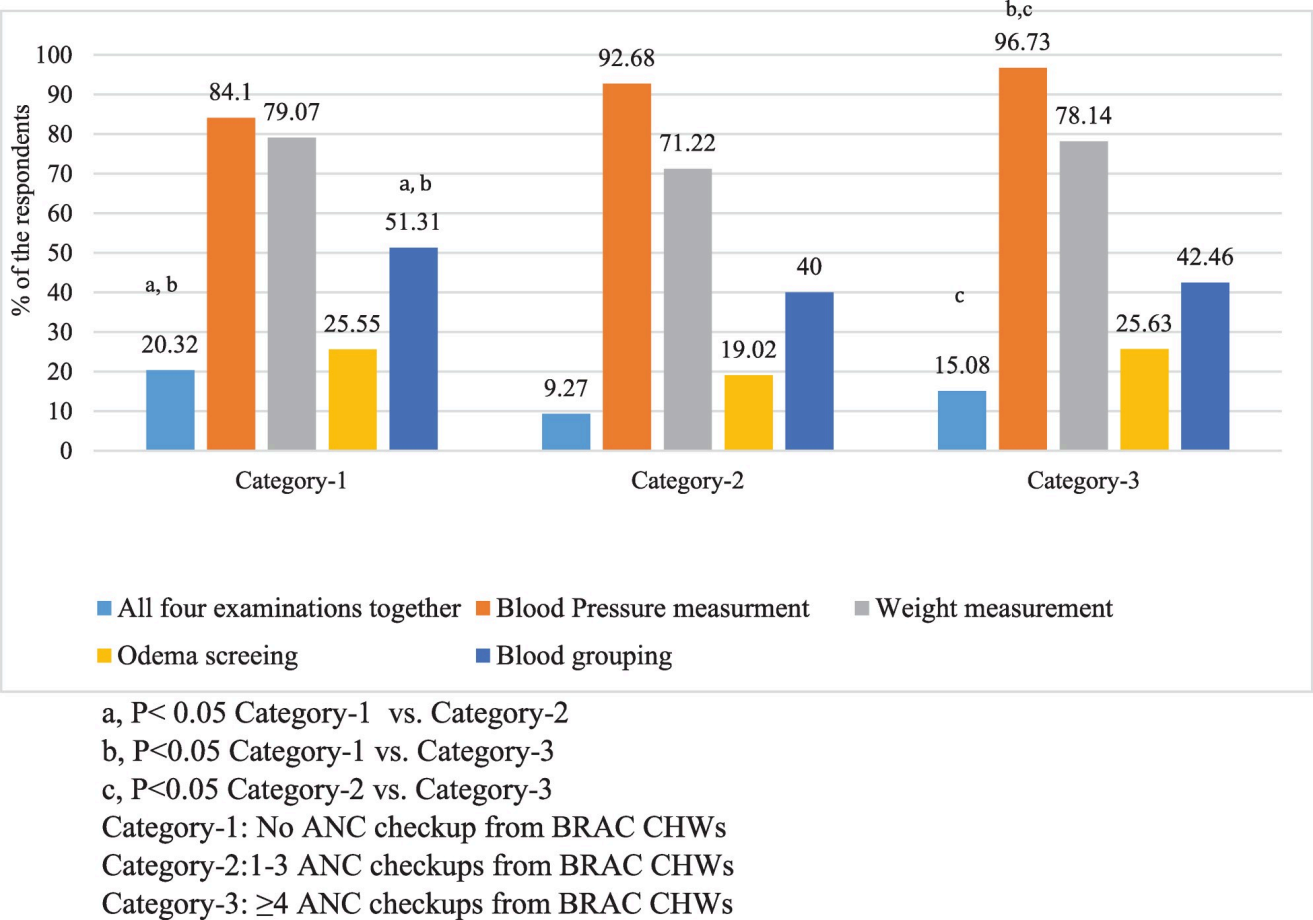
Risk pregnancy screening through weight measurement, odema test, blood grouping, and blood pressure measurement. Fig 2: 1) All four examination together; 2)Blood pressure measurement; 3) Weight measurement; 4) Odema screening; 5) Blood grouping. Fig 2 footnote: a, p< 0.05 Category-1 vs. Category-2. b, p<0.05 Category-1 vs. Category-3. c, p<0.05 Category-2 vs. Category-3. Category-1: No ANC checkup from BRAC CHWs. Category-2: 1–3 ANC checkups from BRAC CHWs. Category-3: ≥4 ANC checkups from BRAC CHWs.

Fig 2 illustrates that in the three categories, a similar proportion of women’s weight was measured and oedema was checked. However, a significantly higher proportion of women’s blood group was examined in category-1 compared to other two categories. We observed that 20.32% women in category-1 received all four tests for identification of risky pregnancy, which was 9.27% and 15.08% respectively in categories 2 & 3 (p<0.05).

Table 3 reveals that having at least four ANC checkups from BRAC CHWs can potentially improve facility delivery by 25% [aPR 1.25; 95% CI (1.01–1.54)]. However, a facility delivery was also found to be associated with the wealth of afamily, ≥four ANC checkups from MTPs and complications during delivery.

**Table 3:** Association between institutional delivery and ANC visits of BRAC CHWs (Cox regression).

Outcome	Predictor	Category	Number of ANC from BRAC CHWs			
			One- three		≥four	
			Category 2		Category 3	
			a PR	95% CI	a PR	95% CI
Institutional delivery ^1^						
	Number of ANC from BRAC CHWs	0 (= 0)	1.00		1.00	
		(= 1)	0.99	0.77–1.28	1.25	1.01–1.54
	Wealth quintile	Poorest (= 0)	1.00		1.00	
		Second (= 1)	1.54	0.98–2.44	1.11	0.78–1.56
		Middle (= 1)	1.65	1.04–2.59	1.41	1.01–1.97
		Fourth (= 1)	1.95	1.25–3.04	1.44	1.03–2.01
		Richest (= 1)	2.03	1.28–3.27	1.50	1.04–2.16
	Year of schooling	None (= 0)	1.00		1.00	
		Primary incomplete (= 1)	0.98	0.63–1.52	0.83	0.59–1.16
		Primary complete (= 1)	1.03	0.65–1.64	0.83	0.58–1.20
		Secondary incomplete (= 1)	1.25	0.84–1.86	1.12	0.83–1.51
		Secondary + (= 1)	1.43	0.92–2.22	1.30	0.91–1.85
	Number of ANC from MTP	<4 (= 0)	1.00		1.00	
		≥4 (= 1)	1.41	1.10–1.82	1.46	1.17–1.83
	Had complication during delivery	No (= 0)	1.00		1.00	
		Yes (= 1)	1.39	1.13–1.71	1.54	1.29–1.84

^1^Model:ANC from BRAC CHWs, parity, types of family, wealth quintile, year of schooling, occupation of the women, religion, distance between hospital and home, four or more ANC from MTPs, and complication during delivery

^a^MTP: Medically Trained Provider includes qualified doctor, nurse, FWV, midwife, paramedics

‘ = 0’- Reference

‘ = 1’–Predictor

‘—‘- Was not in model

aPR- adjusted Prevalence Ratio

CI- Confidence interval

We observed that PNC checkups within 48 hours after delivery from BRAC CHWs was four-fold higher in category-2 [aPR 3.64; 95% CI (1.76–7.54)] and six-fold higher in category-3 [aPR 5.92; 95% CI (3.04–11.53)] compared to category-1 (Table 4). Institutional delivery reduced PNC checkups from BRAC CHWs by 51% in category-3 [aPR 0.49; 95% CI (0.28–0.85)], while, it improved PNC from MTP within 48 hours after delivery among women in category-2 [aPR 15.05; 95% CI (9.46–23.94)] and category-3 [aPR 18.19; 95% CI (11.50–28.77)].

**Table 4:** Association between PNC received by the lactating women within 48 hours and ANC visits of BRAC CHWs (Cox regression).

Outcome	Predictor	Category	Number of ANC from BRAC CHWs			
			One- three		≥four	
			Category 2		Category 3	
			a PR	95% CI	a PR	95% CI
**PNC from BRAC CHWs within 48 hrs after delivery** ^2^	Number of ANC from BRAC CHWs	0 (= 0)	1.00		1.00	
		(= 1)	3.61	1.75–7.47	5.76	2.96–11.20
	Wealth quintile	Poorest (= 0)	1.00		1.00	
		Second (= 1)	0.61	0.22–1.68	0.58	0.28–1.19
		Middle (= 1)	0.66	0.24–1.83	1.16	0.62–2.17
		Fourth (= 1)	0.81	0.29–2.23	0.69	0.31–1.52
		Richest (= 1)	0.67	0.18–2.52	1.15	0.44–3.00
	Year of schooling	None (= 0)	1.00		1.00	
		Primary incomplete (= 1)	1.03	0.40–2.62	0.92	0.48–1.74
		Primary complete (= 1)	1.53	0.57–4.09	0.97	0.47–1.99
		Secondary incomplete (= 1)	1.07	0.42–2.74	0.59	0.28–1.22
		Secondary + (= 1)	----	----	0.27	0.05–1.20
	Number of ANC from MTP^a^	<4 (= 0)	1.00		1.00	
		≥4 (= 1)	0.37	0.12–1.08	1.32	0.63–2.76
	Place of delivery	Home (= 0)	1.00		1.00	
		Institution (= 1)	1.08	0.52–2.26	0.48	0.29–0.81
**PNC from MTP within 48 hrs after delivery**^3^	Number of ANC from BRAC CHWs	0 (= 0)	1.00		1.00	
		(= 1)	1.03	0.81–1.32	1.02	0.82–1.26
	Wealth quintile	Poorest (= 0)	1.00		1.00	
		Second (= 1)	1.10	0.71–1.71	1.09	0.78–1.53
		Middle (= 1)	1.15	0.74–1.78	1.08	0.77–1.50
		Fourth (= 1)	1.15	0.75–1.76	1.07	0.77–1.49
		Richest (= 1)	1.19	0.77–1.85	1.07	0.75–1.53
	Year of schooling	None (= 0)	1.00		1.00	
		Primary incomplete (= 1)	1.09	0.71–1.66	0.99	0.71–1.38
		Primary complete (= 1)	1.06	0.68–1.66	1.03	0.72–1.47
		Secondary incomplete (= 1)	1.11	0.76–1.63	1.04	0.77–1.40
		Secondary + (= 1)	1.07	0.70–1.22	1.02	0.71–1.44
	Number of ANC from MTP^a^	<4 (= 0)	1.00		1.00	
		≥4 (= 1)	0.97	0.77–1.23	1.03	0.83–1.20
	Place of delivery	Home (= 0)	1.00		1.00	
		Institution (= 1)	14.98	9.41–23.84	18.39	11.66–29.00

^2^ Model: ANC from BRAC CHWs, wealth quintile, types of family, year of schooling, occupation of the women, distance between hospital and home, four or more ANC from MTPs, and place of delivery

^3^ Model: ANC from BRAC CHWs, types of family, wealth quintile, years of schooling, occupation of the women, distance between hospital and home, four or more ANC from MTPs, and place of delivery

^a^MTP: Medically Trained Provider includes qualified doctor, nurse, FWV, midwife, paramedics

‘ = 0’- Reference

‘ = 1’–Predictor

‘—‘- Was not in model

aPR- adjusted Prevalence Ratio

CI- Confidence interval

More than four ANC checkups from MTPs and BRAC CHWs could improve practice of seeking treatment against delivery complications by 45% [aPR 1.45; 95% CI (1.06–1.99)], and 40% [aPR 1.39; 95% CI (1.08–1.80)] among women in category-2 and category-3 respectively (Table 5).

**Table 5:** Association between seeking treatment against delivery complication and ANC visits of BRAC CHWs (Cox regression).

Outcome	Predictor	Category	Number of ANC from BRAC CHWs			
			One- three		≥four	
			Category 2		Category 3	
			a PR	95% CI	a PR	95% CI
**Sought treatment against delivery complication**^4^	Number of ANC from BRAC CHWs	0 (= 0)	1.00		1.00	
		(= 1)	1.10	0.81–1.51	1.39	1.08–1.80
	Wealth quintile	Poorest (= 0)	1.00		1.00	
		Second (= 1)	1.03	0.61–1.70	1.04	0.70–1.55
		Middle (= 1)	1.17	0.70–1.95	1.21	0.82–1.82
		Fourth (= 1)	1.33	0.80–2.21	1.53	0.99–2.36
		Richest (= 1)	1.43	0.84–2.44	1.24	0.94–1.65
	Year of schooling	None (= 0)	1.00		1.00	
		Primary incomplete (= 1)	1.19	0.70–2.04	0.75	0.50–1.12
		Primary complete (= 1)	1.17	0.66–2.08	0.87	0.57–1.34
		Secondary incomplete (= 1)	1.39	0.84–2.31	1.02	0.71–1.47
		Secondary + (= 1)	1.20	0.67–2.13	0.87	0.55–1.37
	Number of ANC from MTP^a^	<4 (= 0)	1.00		1.00	
		≥4 (= 1)	1.45	1.06–1.99	0.98	0.66–1.44
	Number of parity	0–1 (= 0)	1.00		1.00	
		2 (= 1)	0.79	0.58–1.06	0.79	0.62–1.02
		≥3 (= 1)	0.70	0.48–1.03	0.62	0.44–0.85

^4^Model: ANC from BRAC CHWs, parity, types of family, wealth quintile, year of schooling, occupation of the women, distance between hospital and home, and four or more ANC from MTPs

^a^MTP: Medically Trained Provider includes qualified doctor, nurse, FWV, midwife, paramedics

‘ = 0’- Reference

‘ = 1’–Predictor

‘—‘- Was not in model

aPR- adjusted Prevalence Ratio

CI- Confidence interval

In addition, institutional delivery could also increase ENC by 33% among neonates in category-2 [aPR 1.33; 95% CI (1.02–1.73)] and 30% in category-3 [aPR 1.30; 95% CI (1.04–1.63)] (Table 6).

**Table 6:** Association between ENC received by the neonates and ANC visits of BRAC CHWs (Cox regression).

Outcome	Predictor	Category	Number of ANC from BRAC CHWs			
			One- three		≥four	
			Category 2		Category 3	
			a PR	95% CI	a PR	95% CI
**Received all ENC after delivery**^5^	Number of ANC from BRAC CHWs	0 (= 0)	1.00		1.00	
		(= 1)	0.85	0.65–1.12	1.11	0.90–1.38
	Wealth quintile	Poorest (= 0)	1.00		1.00	
		Second (= 1)	1.10	0.74–1.63	0.91	0.66–1.25
		Middle (= 1)	1.14	0.76–1.71	0.98	0.71–1.35
		Fourth (= 1)	1.16	0.78–1.74	1.00	0.73–1.40
		Richest (= 1)	1.02	0.67–1.57	0.92	0.63–1.34
	Year of schooling	None (= 0)	1.00		1.00	
		Primary incomplete (= 1)	0.84	0.56–1.25	1.16	0.84–1.60
		Primary complete (= 1)	0.95	0.62–1.46	1.26	0.89–1.78
		Secondary incomplete (= 1)	0.87	0.60–1.28	1.09	0.79–1.50
		Secondary + (= 1)	0.97	0.76–1.26	1.22	0.82–1.80
	Number of ANC from MTP^a^	<4 (= 0)	1.00		1.00	
		≥4 (= 1)	1.04	0.79–1.36	1.09	0.85–1.40
	Place of delivery	Home (= 0)	1.00		1.00	
		Institution (= 1)	1.33	1.02–1.74	1.24	1.002–1.54

^5^ Model: ANC from BRAC CHWs, wealth quintile, types of family, year of schooling, occupation of the women, distance between hospital and home, four or more ANC from MTPs, and place of delivery

^a^MTP: Medically Trained Provider includes qualified doctor, nurse, FWV, midwife, paramedics

‘ = 0’- Reference

‘ = 1’–Predictor

‘—‘- Was not in model

aPR- adjusted Prevalence Ratio

CI- Confidence interval

## Discussion

ANC visits by BRAC CHWs can potentially improve facility delivery in the study areas; though, this is dependent on high intensity of their visits. Wagstaff explained in his conceptual framework that inequality in health seeking behavior were influenced by factors such as, scarce health services, insufficient health financing, weak infrastructure, cultural norms, environment, household practices and social capital [40]. In cities, health facilities are available but, unaffordability, traditional health practices in slum communities and low quality of care for the poor in Government health facilities still remain as demotivating factors [26,27]. In Bangladesh, the Ministry of Local Government has partnership with local NGOs for delivering health care to the urban poor, and this has been proved not to be a sustainable solution for the latter’s dependence on external funding [41]. As a result, an unregulated private and informal sector has emerged to fill this vacuum [41]. A high proportion of delivery at private clinics in this study indicates either the recipients’ inadequate knowledge of affordable BRAC facilities or their preference for C-section delivery. Moreover, their previous unpleasant experience and strong regulation at BRAC facilities for referring cases with obstetric complications to EmOC have led them to avail services at private clinics [42]. Consequently, it has increased the number of C-section deliveries, out-of-pocket expenditure and reluctance in maternal health service utilization [41,43,44]. Therefore, an increased mobility of BRAC CHWs and provision of low cost upgraded delivery facilities for the poor might be a sustainable solution for the slums.

Earlier, maternal health programmes could not achieve equity in providing facility delivery in Bangladesh due to some limitations. One factor to note was that the skilled birth attendant programme had a weak referral linkage and transport facility [42]. The voucher scheme programme improved the use of maternal healthcare services more among the richest than the poor [20,45]. A segment of marginalized people have always been deprived of the benefits of health programmes; however, if they can receive those services, it would be possible to achieve equity [46]. The MANOSHI programme has its own delivery centers, low cost transport facility and strong referral linkages with EmOC to support women with obstetric complications. Community mobilization with high levels of community engagement, health financing and a strong communication between CHWs and potential households through mobile phone would improve their access to care during pregnancy and delivery. As a consequence, it will improve PNC and save lives of both mothers and neonates [13,47–49].

The BRAC CHWs face a constant difficulty in locating pregnant women within their coverage area due to the latters’ circular migration, eviction and long or irregular working hours [41]. Besides, rapid migration from rural areas to urban slums has also added an additional challenge for the CHWs. We observed in our study that women belonging to category-2 had a higher proportion of home delivery than the women in other two categories. However, in terms of receving PNC from BRAC CHWs or MTPs this group has fewer women than the other groups. It seems that these women were outside the catchment area of MANOSHI programme during their pregnancy and delivery period, which created a service gap. Consequently, a superficial knowledge on pregnancy and delivery related care and gap in service utilization exists among those potential beneficiaries [34]. Incidentally, CHWs would not be able to improve knowledge on MNCH care and practice among women, who had migrated into the programme area after becoming pregnant. However, these women still had a scope of being included in the current study. Furthermore, another limitation of CHWs is that they are unable to provide equitable services to beneficiaries and encourage the development of an empowered community to recognize the social determinants of health [50]. Thus, CHWs are needed to be recruited from their native community where they should have a preexisting social relationship; and this will enable them to understand the community obstacles and respond instantly [50]. In addition, provision of home-based free service delivery to the poorest through CHWs with a strong referral system and community sensitization and mobilization are needed [49,50].

Earlier studies found literacy to be one of the predictors of using maternal healthcare services from skilled providers in slum communities of Bangladesh [33] and it is also found to reduce inequity in other parts of the country [45] and abroad [51]. However, this study did not observe any association between years of schooling and use of maternal and neonatal healthcare services. The association between education and health indicators is intuitively reasonable, as educated individuals tend to be more cautious of personal health issues, have higher self-efficacy and exhibit better adherence to self-care and healthy behaviors [52]. Findings of the current study, assumed that increased utilization of maternal health care services might have had an association with literacy but not with length of schooling.

WHO has recommended seeking treatment from EmOC against obstetric complications and acute conditions that lead to maternal death [53]. In the study areas, we observed a tendency of seeking treatment against delivery complications higher than the national average, which has not changed over a decade [54]. The BRAC CHWs counseled women on delivery complications to avail treatment from the nearest EmOC and arrange transport to the facility. Furthermore, ≥four ANC checkups from either MTPs or BRAC CHWs were found to have an association with seeking treatment against delivery complications. All these components supported evidence that ≥four ANC checkups, availability of treatment and a short distance to facility and infrastructure were helpful to identify risky pregnancy and improve delivery at EmOC facilities [55,56]. Similar to findings from earlier studies in Bangladesh, we also found in our study that pregnant women and their family members prefer informal providers as the first point of contact for seeking treatment against delivery complications [42,56]. During the course of informal treatment, when the providers fail, family members of the women bring her to EmOC facilities [42,56]. Therefore, the BRAC CHWs need to build a strong attachment and engagement with the local community by responding appropriately and referring women to either BRAC delivery facilities or EmOC during complications.

Because of the rapid growth of for-profit diagnostic centers and private clinics, women’s preference for ultra-sound has become an integral part of ANC checkup. Respondents who visited MTPs during pregnancy had a higher rate of availing ultra-sound examination as we found in the current study [41,57,58]. As a result, out-of-pocket expenditure of the poor might have increased [41]. Alternatively, it could be interpreted that due to increase in country’s economic growth, capacity of buying MNCH services from the private health sector has been functioning well. Thus, for sustainability of the programme women of this group have to be targeted for BRAC delivery facilities. Overall, findings revealed that the screening of risky pregnancy has reduced due to low frequency of oedema screening. Under reporting by the respondents or ignorance of health care providers on interpreting oedema as a symptom of risky pregnancy might have occurred. ANC checkup along with counseling on the importance of service utilization by BRAC CHWs could improve the continuum of care seeking among their beneficiaries. Consequently, CHWs could serve them better in a timely manner and improve their PNC visits. Low PNC coverage among the women who delivered at facility does not signify that these women did not have an attachment with BRAC CHWs. Since some facilities were not under the working area of BRAC CHWs, they were unable to provide them PNC within expected 48 hours. However, we also observed a gap in PNC visits by BRAC CHWs to the women who delivered at home. This was similar to an earlier study, which means that the trend of low PNC coverage of BRAC CHWs for home delivery has not changed [33]. Perhaps during pregnancy, these women went to parental place for delivery, which is a common practice in Bangladesh [59]. Similar to the MANOSHI programme, BRAC has two other programmes across rural Bangladesh namely, Improved Maternal Neonatal Child Survival (IMNCS) and Essential Healthcare (EHC). Both provide MNCH care services through CHWs. These three programmes could work together in registering and recording information of pregnant women electronically so that with a change of location, local BRAC CHWs would be able to track the pregnant women and provide them the required services. A significant finding in this regards was that women who delivered at a facility had a higher tendency of receiving PNC from MTPs, which supported the evidence that facility delivery confirmed MTP assisted PNC within 48 hours after delivery [60–62].

ANC visits of BRAC CHWs could not ensure ENC; however, institutional delivery could do it. Although, thermal care of the neonates was ensured among all three groups, appropriate cord care was not provided to neonates in categories 2 and 3. Earlier, knowledge gap on ENC practice was also found among slum women [63] and birth attendants might not have enough knowledge to ensure better adherence of ENC at household level [64]. On the other hand, recall bias might have persisted in the responses on ENC, as after delivery, mothers might not have been conscious enough to feel the necessity of ENC components. A higher compliance of ENC in institutional delivery in this study confirmed that both facility delivery and skilled birth attendance were vital for ensuring ENC practices recommended by WHO [65,66].

However, since the current study is a cross-sectional one, it could not show the causal effect of the intervention. During survey, women were asked to recall their last pregnancy, delivery, postpartum and newborn care and the duration of the recall period was three years. Therefore, there might be a recall bias in responses. The respondents were selected from an open cohort and selection was not restricted only to women who were residing in the study areas for more than three years. As a result, service coverage of the MANOSHI programme had a chance to be underestimated and those who came from outside had a chance to receive services from other providers. In addition, not only the BRAC MANOSHI programme was working in the programme areas, but other private and NGO clinics also were there. As a result, we found different public and private providers, which showed that slum people can afford private services and availability of these services made them availed. Furthermore, during door-to-door visit the BRAC CHWs suggested pregnant women for receiving at least one ANC checkup from MTP, birth preparedness, complication readiness and to conduct their delivery by skilled providers. However, to show the association between ANC by BRAC CHWs and safe motherhood and neonatal care, we adjusted for the number of ANC checkup from MTP and found the true effect of ANC by BRAC CHWs. Since the MANOSHI programme has been implemented in slums in all cities of Bangladesh, we could not compare the findings with a ‘control’ city. We conducted a large census and collected samples through randomization. The MANOSHI programme in the 10 study areas was conducted following the same phase but, in BMCs midwivies conduted delivery while in BDCs urban birth attendants conducted the deliveries.

In this study, it has been shown that the ≥four ANC checkups by BRAC CHWs could ensure the facility delivery, PNC and ENC. However, a question remain whether implemention of SE model through BRAC CHWs in MANOSHI programme would be able to serve the poor slum women, benefit them by saving their lives and also make profit for the organization effectively. Pure commercial ventures or for‐profit businesses ensure financial returns and profit, whereas SE needs to fulfill both social and economic returns; thus, the challenges that arise are different compared to commercial businesses [67]. These BRAC CHWs are neither skilled in entrepreneurship nor have received enough training to take their ventures from the philanthropic concept and early stages to grow into large and viable businesses [67]. Thus, the MANOSHI programme needed to raise awareness among the slums people for safe motherhood and ENC. At the same time, this programme needed to provide training to the BRAC CHWs to achieve the SDG-3 as well as making profit for organizational sustainability.

## Conclusion

At least four ANC visits by BRAC CHWs are found to have potential to improve facility delivery among women in BRAC MANOSHI intervention areas. This is expected to lead to an increase in MTP assisted PNC within 48 hours and ENC of neonates. However, the stability in the number of ANC visits of BRAC CHWs seems to reduce inequity in maternal and neonatal health services utilization in different segments of the wealth quintile. Therefore, targeting the poor, compliance with four ANC visits by BRAC CHWs, upgrading BRAC delivery facilities to EmOC and incorporating diagnostic services are urgent for ensuringa safe motherhood and newborn care in slum communities of Bangladesh.

## Supporting information

S1 Data (DTA)
